# A tribute to DNA fingerprinting

**DOI:** 10.1186/2041-2223-4-19

**Published:** 2013-11-18

**Authors:** Manfred Kayser, Antti Sajantila, Bruce Budowle

**Affiliations:** 1 Department of Forensic Molecular Biology, Erasmus MC University Medical Center Rotterdam, Rotterdam, Netherlands; 2 Department of Forensic Medicine, Hjelt Institute, University of Helsinki, Helsinki, Finland; 3 Institute of Applied Genetics, Department of Genetics and Molecular Biology, University of North Texas Health Science Center, Ft. Worth, TX, USA; 4 Center of Excellence in Genomic Medicine Research (CEGMR), King Abdulaziz University, Jeddah, Saudi Arabia

## 

September 2012 marked the 28th anniversary of an experiment that translated into the forerunner of a technology that impacts society worldwide till today, as well as the official retirement of the remarkable scientist who is widely known for this milestone discovery. That is, DNA fingerprinting invented, coined, further developed, and for the first time practically applied by Sir Alec Jeffreys, which changed forensic science for the better and forever. Sir Alec’s fame transcends forensic science, other scientific disciplines, and just as significant the lay public. His invention has promulgated one of the most successful scientific tools in solving and fighting crime, identifying missing persons and victims of mass disasters, and - just as important - in helping exonerate those innocent people who have been wrongly associated with a crime. Although most of his research career was outstanding beyond forensic DNA analysis, Sir Alec is widely considered by the apt moniker “The Founding Father of Forensic DNA Identification”.

To contribute to the affirmation and acknowledgement that Sir Alec’s discovery of DNA fingerprinting has had on a wide range of scientific disciplines, we the Editors-in-Chief, thought it would be important and timely to devote a special series of review articles in *Investigative Genetics* on DNA fingerprinting, its past, present and future. We are pleased to have distinguished experts in forensics, anthropology, zoology, botany, and microbiology who contributed treatises on how Sir Alec’s discovery and subsequent developments of DNA fingerprinting have impacted - and continue to influence - their fields. All these scholars witnessed the nascent days of DNA fingerprinting and used – together with numerous colleagues worldwide - various similarly-based molecular genetic strategies to address a wide range of research questions in their respective fields. Mark Jobling, *Investigative Genetics*’ regular columnist and professor at the same University of Leicester’s Department of Genetics that Sir Alec resided for 35 years, contributed a Guest Editorial to summarize the story of DNA fingerprinting in greater detail.

We remember well the original International DNA fingerprinting Conferences held on five continents from 1990 to 1999 (Bern, Switzerland in 1990; Belo Horizonte, Brazil in 1992; Hyderabad, India in 1994; Melbourne, Australia in 1996; and Port Elizabeth, South Africa in 1999). These conferences, initially organized by Sir Alec and colleagues, were unique and extremely valuable because scientists from different scientific backgrounds were brought together with a common interest in genetically characterizing and differentiating organisms, strains, or species by means of DNA fingerprinting. We recall the lively interactions and how it helped us and many others to gain a foothold in molecular genetics, and at the same time to learn how useful DNA fingerprinting is to address various open questions in the life sciences. Although this International DNA fingerprinting Conference series came to an end within a decade, the science and technology of DNA fingerprinting continued and was rapidly and fully absorbed by various life science disciplines. Predictably there was the necessary end of this exciting conference series; it had served its purpose of establishing DNA fingerprinting as a routine tool across life sciences. Yet, the end was unfortunate because this particular venue for vibrant exchange of science and technology within and across disciplines was gone. Especially in a scientific world where inter-disciplinary work has become increasingly important and at the same time more challenging because of the necessary drive for levels of specialization, it can be difficult to find a common language even among biological scientists.

Sir Alec’s scientific interest in human genomic dynamics was much beyond DNA fingerprinting. In fact, he made numerous groundbreaking discoveries on the processes of mutation and recombination - two important pathways of creating and maintaining genetic diversity - and regularly published them in the highest ranked scientific journals. To keep focus, these notable scientific achievements that likely will provide the fodder for generations of scientists cannot be the target of this special article series; perhaps future ones in this or more specialized journals will be devoted to that part of his enormous research achievements.

We hope that with this special article series on DNA fingerprinting *Investigative Genetics* will attract many readers, some who may read these articles with a nostalgic touch, others to gain a perspective on how a single discovery could have such a strong impact on science and society so quickly as DNA fingerprinting did, and those who seek the motivation to commit themselves to science with the same verve as Sir Alec Jeffreys has.

## Competing interests

The authors declare that they have no competing interests.

## Authors’ contributions

All authors contributed to the manuscript preparation. All authors read and approved the final manuscript.

